# Jasmonic acid is a crucial signal transducer in heat shock induced sesquiterpene formation in *Aquilaria sinensis*

**DOI:** 10.1038/srep21843

**Published:** 2016-02-23

**Authors:** Yan-Hong Xu, Yong-Cui Liao, Zheng Zhang, Juan Liu, Pei-Wen Sun, Zhi-Hui Gao, Chun Sui, Jian-He Wei

**Affiliations:** 1 Institute of Medicinal Plant Development, Chinese Academy of Medical Sciences & Peking Union Medical College, No. 151, Malianwa North Road, Haidian District, Beijing 100193, China; 2 Hainan Branch Institute of Medicinal Plant, Hainan Provincial Key Laboratory of Resources Conservation and Development of Southern Medicine, Chinese Academy of Medical Sciences & Peking Union Medical College, Wanning 571533, China

## Abstract

Agarwood, a highly valuable resinous and fragrant heartwood of *Aquilaria* plants, is widely used in traditional medicines, incense and perfume. Only when *Aquilaria* trees are wounded by external stimuli do they form agarwood sesquiterpene defensive compounds. Therefore, understanding the signaling pathway of wound-induced agarwood formation is important. Jasmonic acid (JA) is a well-characterized molecule that mediates a plant’s defense response and secondary metabolism. However, little is known about the function of endogenous JA in agarwood sesquiterpene biosynthesis. Here, we report that heat shock can up-regulate the expression of genes in JA signaling pathway, induce JA production and the accumulation of agarwood sesquiterpene in *A. sinensis* cell suspension cultures. A specific inhibitor of JA, nordihydroguaiaretic acid (NDGA), could block the JA signaling pathway and reduce the accumulation of sesquiterpene compounds. Additionally, compared to SA and H_2_O_2_, exogenously supplied methyl jasmonate has the strongest stimulation effect on the production of sesquiterpene compounds. These results clearly demonstrate the central induction role of JA in heat-shock-induced sesquiterpene production in *A. sinensis*.

The plants of *Aquilaria* spp. belong to the family Thymelaeaceae, and are typical evergreen trees primarily distributed throughout Southeast Asia. The resinous portion of their branches and trunks, known as agarwood, is widely used in traditional medicine as a digestive, sedative, and anti-emetic and is also popularly used in incense and perfume^1^,^2^,^3^,^4^,^5^,^6^. In the international market, high-quality agarwood is more costly than gold. To date, most agarwood comes from wild *Aquilaria* resources, which has resulted in severe destruction of the natural *Aquilaria* forests in almost all the countries where agarwood has been commercially exploited. To protect wild *Aquilaria* resources and to ensure their sustainability, all species of this genus are listed as endangered species in Appendix II of the *Convention on International Trade in Endangered Species of Wild Fauna and Flora*^7^.

Because only the wounded *Aquilaria* tree can produce agarwood^1^,^8^,^9^,^10^, people have developed various artificial agarwood-inducing methods, such as partly breaking the trunk, making holes with burning chisels and inoculating with fungi^11^. Of these traditional agarwood-inducing methods, only agarwood obtained from holes made with burning chisels meets the requirements in the *Chinese Pharmacopoeia* (2010)^11^,^12^. However, little is known about the signaling pathway and the regulation mechanisms of wound-induced agarwood formation.

The heat shock response is a conserved cellular defense mechanism in response to elevated temperatures and is observed in cells from bacteria to human. Research has indicated that heat shock induces the accumulation of glucosinolates and several other secondary metabolites derived from the phenylpropanoid pathway, including anthocyanins and sinapine derivatives in *Arabidopsis thaliana*^13^. Investigation of *Taxus yunnanensis* cell suspension cultures has shown that heat shock (40 °C to 50 °C for 30 min) can significantly induce paclitaxel production^14^. However, the mechanisms by which heat shock affects secondary metabolite production in *in vitro* cultured plant cells have not been well elucidated.

Jasmonic acid (JA) is a well-characterized long-distance signaling molecule that mediates defensive responses and secondary metabolism in plants^15^,^16^,^17^,^18^. The biosynthesis of JA in plants is essentially a series of enzyme reactions that is initiated by the release of a substrate, α-linolenic acid (LeA), from the cell membrane. LeA is oxidized to 13-(S)-hydrogen peroxide-linolenic acid (13-HPOT) in plastids by lipoxygenases (LOXs), after which catalysis by allene oxide synthase (AOS) and allene oxide cyclase (AOC) produces 12-oxo-phytodienoic acid (12-O-PDA), which enters the cytoplasm. Subsequently, OPDA is reduced by 12-oxophytodienoate reductase (OPR3), followed by three cycles of β-oxidation, resulting in conversion to JA in the peroxisomes^19^,^20^. Thereafter, the volatile methyl jasmonate compound is generated via jasmonic acid carboxyl methyltransferase (JMT). Studies have indicated that expression of these enzymes in the JA biosynthesis pathway greatly affects JA levels in plants. LOX is essential in JA biosynthesis, as different plant species with reduced LOX expression levels have 50–95% reductions in JA levels after wounding^21^,^22^, whereas plants that over-express AOS or AOC constitutively do not exhibit elevated JA levels but show increased JA production after wounding or treatment with other stimuli^23^,^24^,^25^,^26^.

Previous reports have shown that JA plays important roles in sesquiterpene biosynthesis. In rice, MeJA markedly induced the expression of sesquiterpene synthase *TPS3* and the release of more than 10 sesquiterpenes, particularly (E)-β-caryophyllene *(Oryza sativa)*^27^. Meas *et al*. showed that exogenous JA treatments promoted expression levels in the artemisinin biosynthetic pathway, ultimately leading to increased artemisinin accumulation in *A. annua*^28^. The induction role played by JA in *Aquilaria* spp. has also been widely demonstrated in recent years. Exogenously applied MeJA in *Aquilaria* cell suspension cultures or calluses induced biosynthesis and accumulation of sesquiterpenes compounds, especially δ-guaiene^2^,^6^,^29^,^30^. However, whether these results are directly related, the significance and function of endogenous JA in the agarwood sesquiterpene biosynthetic pathway remains unknown.

In the present study, we treated *A. sinensis* cell suspension cultures with heat shock, imitating the burn-chisel-drill method used on trees, to investigate how JA affects the accumulation of sesquiterpene compounds. We found that endogenous JA and its methyl ester accumulate rapidly and transiently after heat shock treatment. Correspondingly, the expression of genes in the JA biosynthesis pathway was significantly up-regulated, and sesquiterpene compounds accumulated. A specific inhibitor of JA, nordihydroguaiaretic acid (NDGA), could block these effects. Additionally, when exogenously supplied to *A. sinensis* cells, methyl jasmonate exhibited the strongest effect on sesquiterpene biosynthesis compared to SA and H_2_O_2_. These data demonstrate that JA is a critical signal transducer in the intracellular signal cascade induced by heat shock and that JA ultimately plays a role in the accumulation of sesquiterpene compounds.

## Results

### Heat shock treatment induces expression of a set of genes involved in JA biosynthesis, perception and transduction pathways

**Figure 1:**
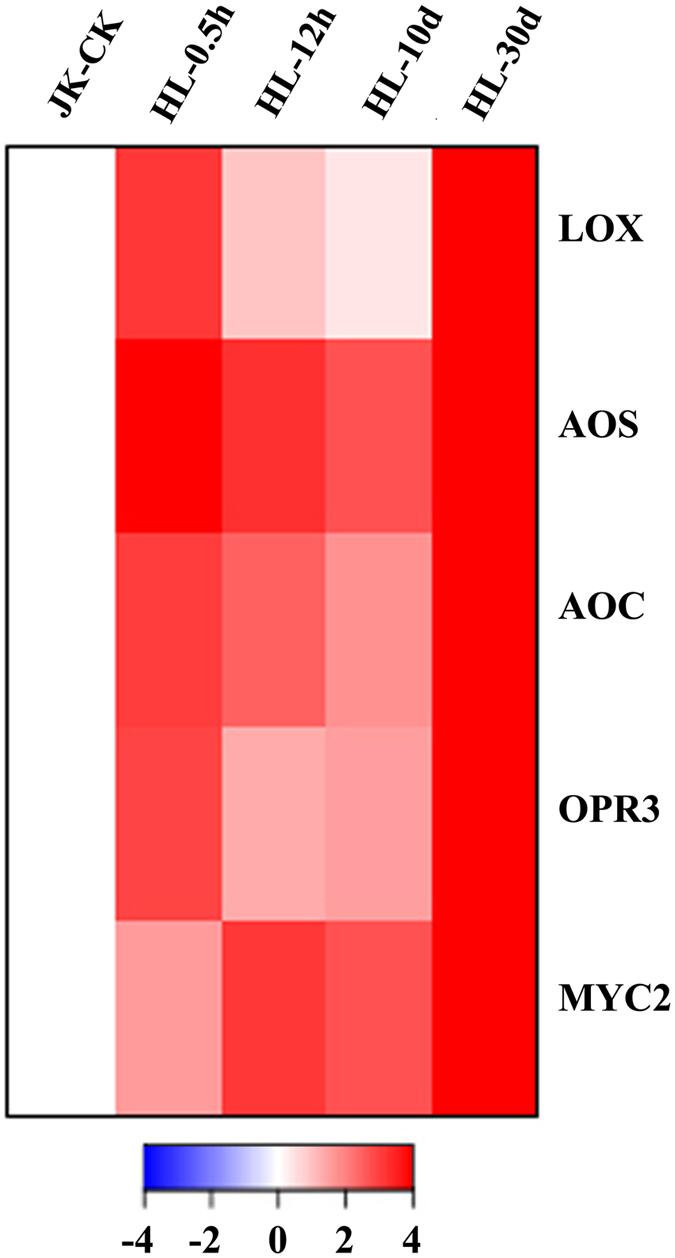
Heatmap of gene expression levels. This heatmap is based on hierarchical clustering (*P *>* *0.001) of genes involved in JA biosynthesis and regulation. All gene expression levels were transformed to scores ranging from −4 to 4 and were colored blue, white, or red to represent low, moderate, or high expression levels, respectively. The relative expression levels were scaled based on their mean and do not represent expression levels in comparison with controls.

Making holes in *A. sinensis* using a burning chisel is a traditional agarwood-inducing method, that agarwood obtained by which could meet the requirements in the *Chinese Pharmacopoeia* (2010)^11^,^12^. We used Solexa technology to sequence *A. sinensis* materials, in which holes had been made using a burning chisel, so that profiling of gene expression levels could be accomplished (unpublished data). Based on the results obtained, this treatment positively induced the expression of genes involved in JA biosynthesis, including lipoxygenase (LOX), allene oxide synthase (AOS), allene oxide cyclase (AOC) and 12-oxophytodienoate reductase 3 (OPR3). After treatment for 0.5 h, the expression levels of these genes were significantly increased, with fold increases of 23.3, 58.4, 21 and 18.5, respectively. The heat map generated by the hierarchical clustering of genes associated with the JA biosynthesis pathway is shown in Fig. 1. Note that the heat map displays relative expression levels across all the samples analyzed rather than absolute expression levels for each sample. These results led to the hypothesis that JA plays a role in the effects of heat shock treatment.

**Figure 2:**
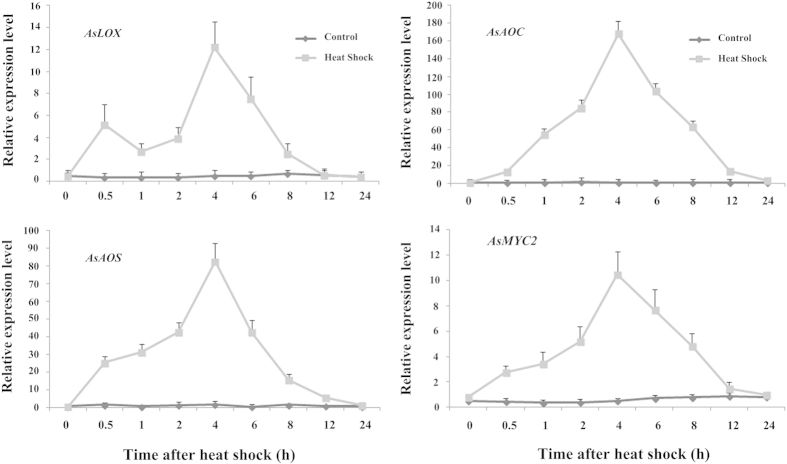
Expression levels of genes after heat shock treatment. Cultures of *A. sinen*sis cells in suspension were subjected to heating to 50 °C for 30 min. After this heat shock treatment, the culture flasks were returned to the gyratory shaker at 25 °C for continued shaking and were sampled at appointed times (0 h, 0.5 h, 2 h, 4 h, 6 h, 12 h, 24 h, and 3d). Healthy cells in suspension culture that were not subjected to heat shock treatment were used as controls. Expression levels of *AsLOX*, *AsAOS*, *AsAOC* and *AsMYC2* were assayed using real-time PCR analysis and *AsGADPH* as the internal control. All genes were up-regulated in response to heat shock treatment. Each value is the mean ± SE of 3 independent biological replicates.

To test this hypothesis, we subjected *A. sinensis* cell suspension cultures to heat shock treatment, imitating the burn-chisel-drill method utilized on trees, to evaluate the expression levels of genes involved in the JA biosynthesis pathway. As cell suspensions have been reported to exhibit a certain degree of thermo tolerance, cell viability was only affected by temperatures higher than 40 °C; however, cell viability declined significantly at these higher temperatures, and the cells did not exhibit any capacity for growth recovery at temperatures >50 °C^14^,^31^,^32^,^33^. The heat shock experiment was performed by placing *A. sinensis* cell suspensions in a 50 °C water bath for 30 min, after which samples were acquired at appointed times for analysis. This treatment induced the expression of *LOX*, *AOS* and *AOC*, which showed similar expression profiles (Fig. 2). Expression of *AOS* and *AOC* increased gradually from 0.5 h to 4 h after heat shock treatment, with expression levels increasing 80- and 160-fold, respectively, peaking at 4 h, and subsequently declining gradually within 24 h. By contrast, *LOX* showed two peaks at 0.5 h and 4 h, peaking at 4 h with a 12-fold increase. This result is consistent with the conclusions of previous studies that the regulation of JA biosynthesis is determined by a positive feedback loop and substrate availability^34^,^35^,^36^,^37^. When plants are injured, genes involved in JA synthesis are activated, inducing the biosynthesis and accumulation of JA compounds. Correspondingly, wound-induced JA compounds activate the expression of JA biosynthesis genes. This feedback may explain why *LOX* expression after heat shock treatment exhibits two peaks (Fig. 2). Additionally, the expression profile of *MYC2*, as a master regulator of most aspects of the JA signaling pathway^38^,^39^,^40^, is similar to those of *AOS* and *AOC* (increasing from 0.5 h to 4 h and then declining gradually within 24 h), demonstrating that the JA signaling pathway is activated via the heat shock process.

### Endogenous jasmonic acid and its methyl ester increase rapidly after heat shock, while NDGA blocks it

**Figure 3:**
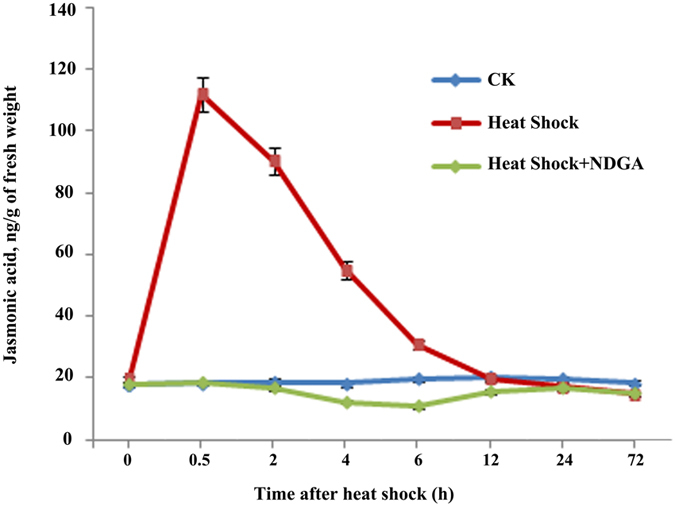
Time course of endogenous jasmonates induced by heat shock treatment. *A. sinensis* cells cultured in suspension containing or not containing 1.0 μM nordihydroguaiaretic acid (NDGA), an inhibitor of JA biosynthesis, were subjected to heating at 50 °C for 30 min. After the heat shock treatment, the culture flasks were returned to the gyratory shaker at 25 °C for continued shaking and were sampled at appointed times (0 h, 0.5 h, 2 h, 4 h, 6 h, 12 h, 24 h, and 3d). Healthy cells cultured in suspension that did not receive heat shock treatment were used as controls. Endogenous jasmonates were extracted and analyzed by GC-MS. Heat shock treatment rapidly induced JA production in *A. sinensis* cell suspensions, and NDGA, a specific inhibitor of JA, could block this induction, as demonstrated by the lack of JA accumulation. CK indicates the healthy control; Heat Shock indicates NDGA-free treatment in a 50 °C water bath; Heat Shock + NDGA indicates NDGA treatment in a 50 °C water bath.

Studies have shown that jasmonate is rapidly synthesized only in response to external stimuli, such as treatments with fungi, pathogens, insects or herbivory^41^,^42^,^43^,^44^,^45^. Furthermore, the wound-induced rise in JA is transient and appears before the expression of *LOX*, *AOS* and *AOC*^46^,^47^. To investigate whether heat shock promotes the production of endogenous JA, the JA contents of three samples (healthy control, heat shock, and heat shock plus NDGA) were determined using GC-MS. As shown in Fig. 3, after heat shocking the cell suspension cultures in a 50 °C water bath, a transient increase in the concentration of JA was observed, which peaked 30 min after heat shock treatment with a value of 112.09 ng/g [fresh weight], and approximately 6-fold increase compared with the healthy control (no heat shock). Subsequently, the JA concentration gradually declined to a normal level at 12 h, indicating that the induction of JA results in rapid transient accumulation after heat shock and is, therefore, an early defense response. By contrast, almost no difference was observed between healthy (no heat shock treatment) and JA-inhibitor-containing cell cultures.

### Sesquiterpene compounds accumulate after heat shock, whereas NDGA significantly inhibits sesquiterpene production

**Figure 4:**
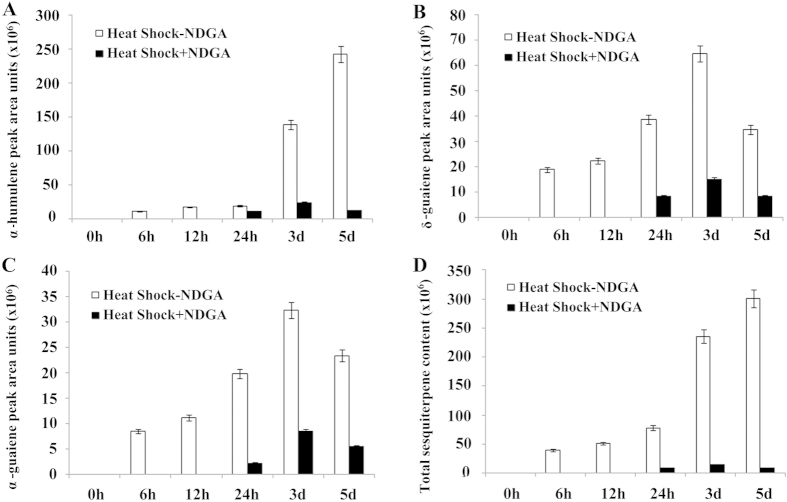
Changes in sesquiterpene compounds in heat-shock-treated cells in suspension. The fused silica fiber of the Solid Phase Micro Extraction (SPME) was introduced into the headspace above the ground samples (4-g each), where this fiber adsorbed volatile components for 30 min. The adsorbed components were analyzed by GC-MS. The relative contents of sesquiterpene compounds were reflected by the peak areas generated. (**A**) Relative amount of α-humulene at different time points, as determined by peak areas. (**B**) Relative amount of δ-guaiene at different time points, as determined by peak areas. (**C**) Relative amount of α-guaiene at different time points, as determined by peak areas. (**D**) Total amount of sesquiterpene compounds obtained after heat shock treatment or heat shock treatment with NDGA. The hollow column indicates heat shock samples, while the solid column indicates heat shock samples containing NDGA.

To confirm the role of endogenous JA in heat-shock-induced agarwood formation, volatile oils were extracted from the three samples (healthy control, heat shock, heat shock plus NDGA) via a solid-phase micro-extraction method and were analyzed by GC-MS. No sesquiterpene compounds were detected in the healthy control sample (Supplementary Fig. S1), whereas three sesquiterpene compounds were detected in the heat shock samples, (Supplementary Fig. S2A,B). Of these, the α-humulene content was the highest (Fig. 4A), followed by δ-guaiene and α-guaiene (Fig. 4B,C). Interestingly, these were all induced beyond 6 h after heat shock treatment, and their relative contents increased smoothly until 72 h. Maximum δ-guaiene and α-guaiene contents were obtained 3 d after heat shock treatment, whereas the maximum α-humulene content was reached 5 d after heat shock treatment, with a 19-fold increase compared with the control sample. By contrast, very little volatile oil content was detected beyond 24 h after heat shock treatment in the samples containing NDGA. The total sesquiterpene content in the heat shock samples increased 9-fold at 24 h, 15-fold at 3 d, and 35-fold at 5 d compared with that of the NDGA-containing samples (Fig. 4D), clearly demonstrating that production of endogenous JA plays a critical role in heat-shock-induced sesquiterpene production. It is noteworthy that, although we observed no increase in JA levels in the NDGA-containing samples until 72 h after heat shock treatment (Fig. 3), volatile oils were still detected 24 h after heat shock treatment. A possible explanation for this phenomenon is that the biosynthesis of sesquiterpene is not solely dependent on the JA signaling pathway. Although JA generation is completely inhibited or does not accumulate to detectable levels within 24 h after heat shock treatment, other signaling molecules may function alongside sesquiterpene production in a defensive response.

### Exogenous MeJA affects the production of agarwood sesquiterpene more strongly compared to SA and H_2_O_2_

To further demonstrate that the production of JA and methyl jasmonate triggers the biosynthesis of sesquiterpene defense compounds in plant cell suspension cultures, we treated *A. sinensis* calluses with 100 μM MeJA, SA or H_2_O_2_ to examine the effects of exogenous signal molecules on the production of violate oils. GC-MS analysis (Supplementary Fig. S3) revealed that the species and amounts of violate ingredients change greatly in both healthy and treated samples, with alkanes comprising an abundant proportion of the volatile oils in the healthy samples, whereas treated samples were rich in sesquiterpenes and aromatic constituents (Table 1). The peak areas indicated relative contents, with the healthy sample weighing in at 14.512, whereas the values for the MeJA-, H_2_O_2_- and SA-treated samples were 905.9, 383.95 and 85.24, respectively. Production of sesquiterpene compounds (β-elemene, β-humulene, α-guaiene, α-humulene, δ-guaiene) significantly increased in response to MeJA, H_2_O_2_ and SA treatments, with the greatest increasing observed for MeJA, followed by H_2_O_2_ (SA exhibited the weakest induction). It is noteworthy that of these sesquiterpene compounds, β-elemene, α-guaiene and δ-guaiene are also products of sesquiterpene synthase ASS1 catalysis^6^; meanwhile, α-humulene, δ-guaiene and α-guaiene are induced by heat shock treatment (Fig. 4), demonstrating the crucial role of JA in inducing the biosynthesis of agarwood sesquiterpene.

## Discussion

Numerous studies have shown that mechanical wounds, chemical wounds, and fungal infection can induce the formation of agarwood in *A. sinensis*^8^,^9^,^10^,^48^,^49^,^50^,^51^, and agarwood is widely used in traditional medicine, incense, and perfumes across Asia, the Middle East, and Europe^1^,^2^,^3^,^4^,^5^,^6^. Despite the economic and pharmacological value of agarwood, studies regarding the mechanism by which such injuries induce agarwood formation are rare.

A previous study in our laboratory showed that holes made by a burning chisel in an *A. sinensis* tree can produce high-quality agarwood that meets the requirements of the *Chinese Pharmacopoeia* (2010)^11^,^12^. From the Solexa analysis, we found that expression levels of genes involved in JA biosynthesis and regulation pathways were almost up-regulated by heat shock wounding (Fig. 1), indicating that JA may act as a signal molecule. It was proposed the effect of heat shock is to initiate the JA synthesis pathway, which generates a long distance transport of signaling molecules, maybe JA itself or its derivatives.

Previous studies have shown that JA is involved in fungus-induced secondary metabolism^41^,^52^,^53^,^54^. The present study provides evidence that JA plays a critical role in regulating heat-shock-induced agarwood sesquiterpene formation, in agreement with previous studies. The results herein demonstrate that endogenous JA and its methyl ester accumulate rapidly and transiently after *A. sinensis* cell suspensions are treated with a 50 °C water bath for 30 min (Fig. 4). Correspondingly, we monitored three mainly sesquiterpene compounds 6 h after heat shock treatment and found that they accumulated within 72 h (Fig. 4). A specific inhibitor of JA, NDGA, could block the JA signaling pathway and markedly reduce the accumulation of sesquiterpene compounds (Figs 3 and 4), clearly demonstrating that sesquiterpene formation is JA-dependent. Correspondingly, the expression levels of the principle genes involved in JA biosynthesis (*LOX*, *AOS* and *AOC*), which are located in a companion cell-sieve element complex^55^,^56^, were up-regulated after heat shock treatment (Figs 1 and 2). Based on these findings, it is possible that heat shock triggers the synthesis and accumulation of JA, which is mobile within the phloem and becomes a secondary signal that induces the expression of defense genes. However, this induction of defense genes depends on JA perception and transduction but not the capacity of JA synthesis. In support of our hypothesis, MYC2, a master of JA signaling, was also up-regulated (Figs 1 and 2). Studies have shown that JA-induced biosynthesis of nicotine in tobacco^57^, sesquiterpene in *Arabidopsis*^58^,^59^, and alkaloid in *Catharanthus roseus*^60^,^61^ are all mediated by transcription factor MYC2. However, whether the regulation mechanism in *A. sinensis* is consistent with these other studies is unclear and requires further study. We now know that this elicitation process occurs, with gene activation ultimately leading to the synthesis of JA and the activation of the JA signaling pathway. JA and its derivatives have an integral role in the cascade of events that occur during the elicitation process, either directly or indirectly activating the genes involved in sesquiterpene biosynthesis. This explains how agarwood can be induced in an entire tree rather than being formed only in proximity to an injured site.

It is noteworthy that even though gene expression and JA production in the samples containing the JA inhibitor were hardly changed compared with the healthy control sample, violate oils still accumulated to a small degree in the former (Fig. 4). These results imply that, although heat-shock-induced sesquiterpene biosynthesis is JA-dependent, other signal molecules may also be involved in this process. A burst of reactive oxygen species (ROS) is a common event in both biotic and abiotic induced synthesis and the accumulation of secondary metabolites, the wound signaling of which H_2_O_2_ has been proposed to play a role^62^,^63^,^64^,^65^. The phytohormones SA and JA are also recognized play crucial roles in regulating the defensive signaling network^62^,^66^,^67^. More importantly, studies have shown that agarwood sesquiterpene production is induced by these signaling molecules. For instance, H_2_O_2_ induced the formation of vessel occlusions and sesquiterpene as a result of pruning^68^, and Liu *et al*. found that H_2_O_2_ promoted programmed cell death and SA accumulation during the induced production of sesquiterpenes in cell suspension cultures of *A. sinensis*^69^. SA induced the production of three sesquiterpene compounds (α-guaiene, α-humulene and δ-guaiene) in *A. sinensis* cell suspensions^2^, and MeJA induced the expression of sesquiterpene synthase and produced three sesquiterpenes in *A. sinensis* cell suspension cultures or calluses^2^,^3^,^6^,^29^. However, the specific roles of these compounds are not fully understood.

In the present study, we compared the effect of exogenously applied JA with those of SA and H_2_O_2_ on the induction of agarwood sesquiterpene biosynthesis, and our results agree with previous reports^2^,^6^,^29^,^30^,^68^,^69^. All of them induced the accumulation of sesquiterpene compounds, but MeJA exhibited the strongest induction role, with an effect that was 3-times greater than that of H_2_O_2_ and more than 10-times greater than that of SA. These results clearly demonstrate the crucial role played by JA in sesquiterpene production.

The results of the present study also demonstrate that sesquiterpene production is dependent on the endogenous production of JA and is strongly elicited by exogenously applied JA. Thus, the experiments herein have identified JA as a crucial signal transducer in heat-shock-induced agarwood formation. This information facilitates our understanding of the potential mechanism by which wounds induce the formation of high-quality agarwood and provide new clues for improving agarwood-inducing techniques.

## Materials and Methods

### Plant materials and treatments

Cell suspensions of *A. sinensis* were grown in Murashige-Skoog media on a gyratory shaker (110 rpm) at 25 °C in the dark for 15 days prior to heat shock treatments. All heat shock experiments were carried out in a water bath with a removable shaking carriage (DSHZ-300A, China). Cell suspensions with and without 1.0 μM nordihydroguaiaretic acid (NDGA), an inhibitor of JA biosynthesis, were subjected to a water bath at 50 °C for 30 min. After heat shock treatment, the culture flasks were returned to the gyratory shaker at 25 °C and shaking was continued. The cultures were then sampled at pointed times (0 h, 0.5 h, 2 h, 4 h, 6 h, 12 h, 24 h, and 3 d). After filtering with a 100-mesh filter and gassing with a pump, the samples were immediately frozen in liquid nitrogen and then stored at −80 °C. For every treatment, three independent repetitions of the biological experiments were performed.

### Determinations of endogenous jasmonate

Endogenous JA was determined according to the method of Zhang *et al*.^70^. Four grams of cells sampled from *A. sinensis* cell suspension cultures were ground in liquid nitrogen and then transferred to a 15-ml Eppendorf tube containing 7 ml of 80% methanol, to which was immediately added 100 ng of 9,10-dihydrojasmonic acid (DHJA), which was synthesized from JA or its methyl ester by catalytic hydrogenation with Pd/charcoal, as internal standards and 1.0 mg diethyldithiocarbamate (DDTC) as antioxidant. The samples were then extracted at −20 °C overnight. After vacuum filtration, the samples were frozen and thawed three times and then centrifuged for 20 min at 12,000 rpm. The supernatant was stirred with non-soluble polyvinyl pyrrolidone. Subsequently, samples were extracted three times with ethyl acetate, after which they were evaporated at 35 °C to dryness. The residues were dissolved in 100 μL of methanol, and an excess of diazomethane in ether (1 mL) was added. After 30 min at room temperature, the sample was transferred to a capillary. Sample aliquots of 3 μL were analyzed by gas chromatography/mass spectrometry (GC/MS) (Agilent 7890BGC-5977AMS) under the following conditions: linear He flow at 23 cm/s; column temperature gradient: 50 °C for 1 min, 50–160 °C at 30 °C/min, 160–200 °C at 5 °C/min, 200–290 °C at 30 °C/min, 290 °C for 5 min; electron potential, 70 eV. Methyl jasmonate content was determined based on the relationship by which the ratio of the peak area equals the concentration ratio: N_JA_ = (0.1542 × A_224_/A_226_ + 0.0146) × N_DHJA_. N_JA_ and N_DHJA_ represent the concentrations of JA and DHJA, respectively; A_224_ and A_226_ represent the peak areas of JA (224 m/z) and DHJA (226 m/z), respectively.

### GC-MS analysis of sesquiterpene components in *A. sinensis* cell suspensions and calluses

GC-MS analysis was performed using a Varian 450 GC (USA) equipped with a VF-5MS capillary column (internal diameter, 30 m × 0.25 mm; film thickness, 0.25 μm), and a Varian 300 mass spectrometer with an ion-trap detector in full-scan mode under election impact ionization (70 eV). The carrier gas was helium, and the flow rate was 1 mL/min. The injections were performed in splitless mode at 250 °C. Samples were powdered, requiring the same weight for placement into the 12-mL sample bottle, and balanced for 30 min in 60 °C water. The fused silica fiber used for Solid Phase Micro Extraction (SPME) was introduced into the headspace above the sample, and volatile components were adsorbed for 30 min, followed by a thermal desorption process in which the SPME fiber was introduced into the injection port of the gas chromatographic system. The program was immediately started, and the fiber was removed after 10 min. The GC-MS conditions and identification of sesquiterpenes compounds pertained to previously described procedures^4^. The two fibers were injected separately and run in the same program, and relative components were obtained via normalization of peak areas without applying correction factors. We did not use an internal standard because of the difficulty in choosing one that was suited for both samples. Peaks areas pertained to the amounts of compounds in the two different samples to some degree, as the weight of the two samples and the analytical methods were identical.

### Quantitative real-time PCR (RT-PCR) analysis

For To analyze gene expression levels, total RNA was isolated from treated cell suspensions or calluses. A 2-μg aliquot was subjected to first-strand synthesis using M-MLV reverse transcriptase (Promega, USA) and an oligo (dT18) primer. The suitabilities of the oligonucleotide sequences, in terms of annealing efficiencies, were evaluated in advance using the Primer 5.0 program. A fragment of the glyceraldehyde-3-phosphate dehydrogenase (GADPH) gene^71^ was also amplified as an internal control. The qRT-PCR analysis was performed using a BioRad Real-Time System CFX96TM C1000 Thermal Cycler (Singapore). Amplification of the target genes was monitored during every cycle by detecting SYBR-green fluorescence. The Ct (threshold cycle), defined as the PCR cycle at which a statistically significant increase in reporter fluorescence was first detected, was used to measure the starting copy numbers of each target gene. Relative quantitation of each target gene expression level was performed using the comparative Ct method^72^ (comparing the CTs of the target genes with that of the housekeeping gene using 2^−ΔΔCT^). All experiments were repeated at least 3 times, such that there were 3 independent repetitions of each biological experiment. The primers used in this study are listed in Supplementary Table S1.

## Additional Information

**How to cite this article**: Xu, Y.-H. *et al*. Jasmonic acid is a crucial signal transducer in heat shock induced sesquiterpene formation in *Aquilaria sinensis*. *Sci. Rep*. **6**, 21843; doi: 10.1038/srep21843 (2016).

## Supplementary Material

Supplementary Information

## Figures and Tables

**Table 1:** Violates components in different treated samples.

T_R_ Sesquiterpene and aromatics	Compounds	RI	Peaks areas (x10^8^)			
			Healthy	H_2_O_2_ treated	MeJA treated	SA treated
12.209	β-elemene	1198.425	–	7.89	39.89	–
12.808	β-humulene	1212.378	–	2.73	7.94	–
13.008	α-guaiene	1217.037	5.52	109.3	248.6	17.5
13.483	α-humulene	1228.101	–	13.29	86.96	12.73
13.583	Naphthalene	1230.431	3.01	5.07	6.75	1.39
14.161	Bicyclo[5.3.0]decane	1243.894	–	2.56	8.66	–
14.461	δ-guaiene	1250.883	5.95	242.7	507.1	53.62
Total peak areas			14.512	383.95	905.9	85.24
Alkanes						
10.774	Tridecane	970.216	2.41	0.939	1.75	1.02
12.196	Tetradecane	1198.122	7.36	–	–	0.672
14.056	Pentadecane	1241.449	11.13	4.63	14.02	3.99
16.776	Hexadecane	1626.551	4.58	2.46	9.39	1.81
20.994	Heptadecane	1668.993	3.73	–	7.05	–
Total peak areas			29.21	8.029	32.21	7.492

‘TR’ indicates retention time; ‘RI’ indicates retention indices which were calculated against C_8_–C_40_ *n*-alkanes on the non-polar VF-5MS column; ‘–’ indicates not detected.
