# Supplementary material for: Jasmonic acid is a crucial signal transducer in heat shock induced sesquiterpene formation in Aquilaria sinensis
Source: Sci Rep. 2016 Feb 23;6:21843. doi: 10.1038/srep21843 (PMC4763180; doi:10.1038/srep21843)

Jasmonic acid is a crucial signal transducer in heat shock induced sesquiterpene formation in *Aquilaria sinensis*

Running title: Jasmonic acid and agarwood sesquiterpene in *Aquilaria sinensis*

Yan-hong Xu[1,*], Yong-cui Liao[1,*], Zheng Zhang[1,2], Juan Liu[1], Pei-wen Sun[1], Zhi-hui Gao[1], Chun Sui[1], Jian-he Wei[1,2,§]

[1]Institute of Medicinal Plant Development, Chinese Academy of Medical Sciences & Peking Union Medical College, No. 151, Malianwa North Road, Haidian District, Beijing 100193, China

[2]Hainan Branch Institute of Medicinal Plant (Hainan Provincial Key Laboratory of Resources Conservation and Development of Southern Medicine), Chinese Academy of Medical Sciences & Peking Union Medical College, Wanning 571533, China

*These authors contributed equally to this work.

[§]Corresponding author: E-mail, wjianh@263.net; Fax, 86 10 57833358

Supplementary Data

Supplementary Table 1. Primers used in this study.

Gene	Primer name	Sequence (5′- 3′)
LOX	*LOX*-f	GTGGTTGCGAGATGACGAA
	LOX-r	CAAGGAATGGCAGGTAGATG
AOS	*AOS*-f	GTTCAAACACTCAGGGATGC
	AOS-r	AAAAGAGACGCTCGGCTAC
AOC	*AOC*-f	AGCTCTTCTACACCTTCTACC
	AOC-r	GCCTGCTTCTATCACAATCA
MYC2	*MYC2*-f	AGTGATCCTGCCACCGCAG
	MYC2-r	GGCCTATTGTTACTCTCCAG
Internal control	*GAPDH-f*	CTGGTATGGCATTCCGTGTA
	GAPDH-r	AACCACATCCTCTTCGGTGTA

Supplementary Fig S1. Gas chromatography-mass spectroscopy profiles of sesquiterpene compounds in healthy control sample. Total ion chromatogram of the products from healthy *A. sinensis* suspension cells.

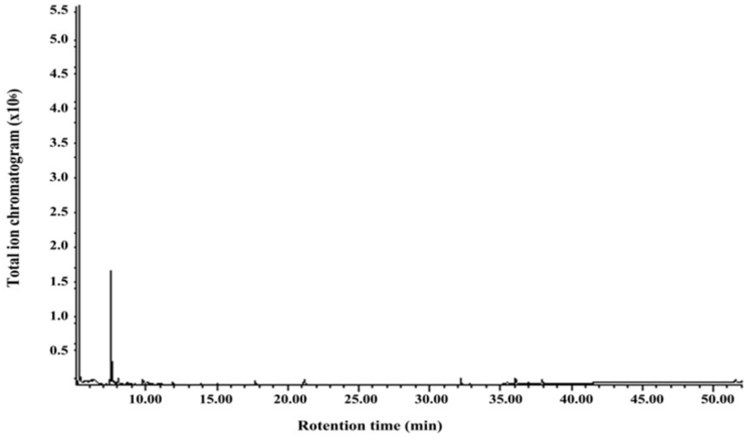

Supplementary Fig S2. Gas chromatography-mass spectroscopy profiles of sesquiterpene compounds in healthy control and heat-shock-treated sample. (A) Total ion chromatogram of the products from heat shock treated *A. sinensis* suspension cells. (B) Mass spectra of the sesquiterpenes and their authentic standards. Peak 1 is α-guaiene, peak 2 is α-humulene, and peak 3 is δ-guaiene.

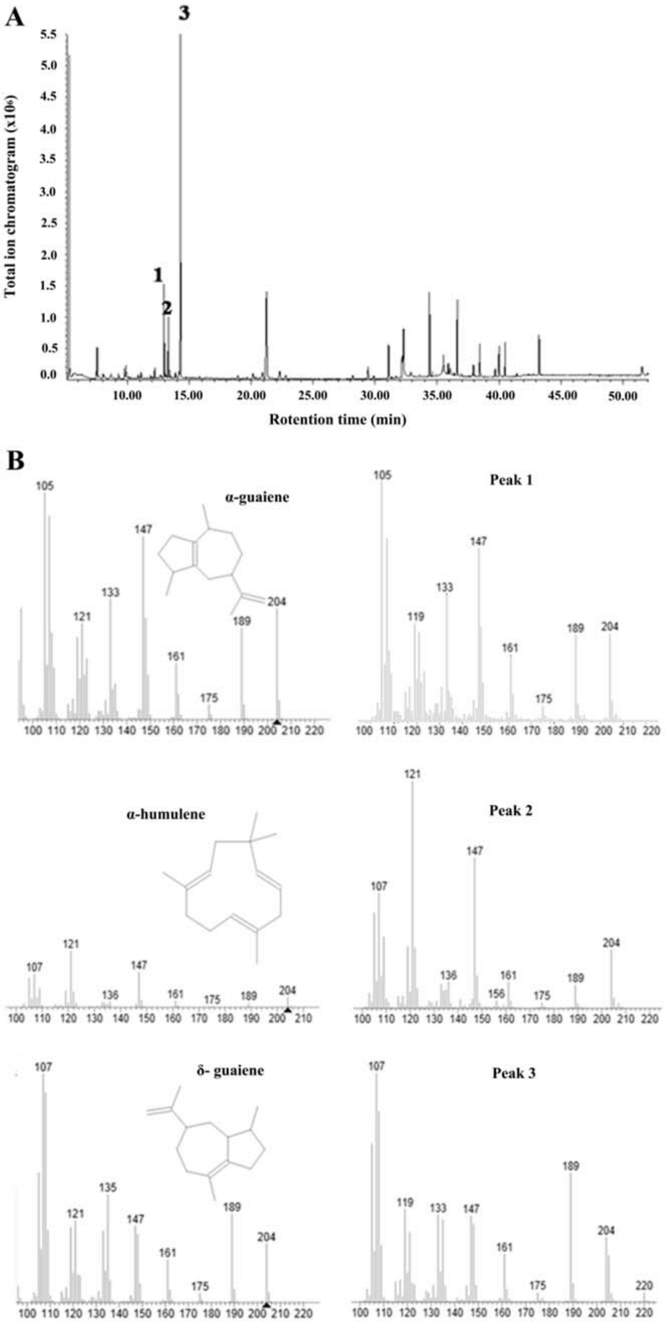

Supplementary Fig S3. GC chromatograph of essential oil of agarwood in healthy and treated samples. 1- Control, healthy sample; 2- H_2O_2 treated sample; 3-MeJA treated sample; 4-SA treated sample.

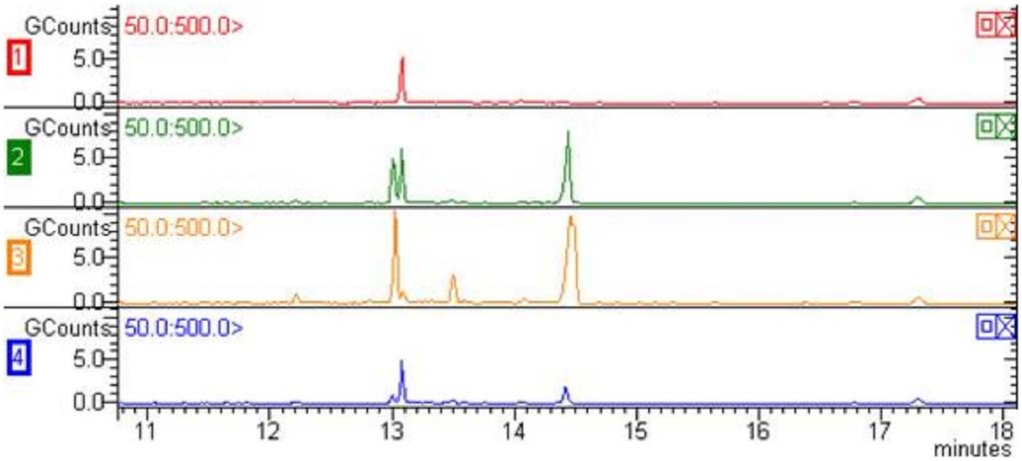

Supplement: Supplementary Information [file srep21843-s1.pdf]
